# Psychological Support to the Community During the COVID-19 Pandemic: Field Experience in Reggio Emilia, Northern Italy

**DOI:** 10.3389/fpsyg.2020.561742

**Published:** 2020-11-17

**Authors:** Fiorello Ghiretti, Gabriela Gildoni, Gaddo Maria Grassi, Laura Torricelli, Elena Benassi, Elisa Bonaretti, Francesca Bonazzi, Sara Borelli, Francesca Cagnolati, Katia Covati, Francesca Errera, Vanessa Finardi, Rossano Grisendi, Jody Libanti, Roberta Lumia, Annachiara Montanari, Giorgia Morini, Sabrina Pettinari, Annamaria Peverini, Caterina Ragone, Marco Santachiara, Valerio Valentini, Agnese Zanchetta, Sabina Zapponi, Luana Pensieri, Michele Poletti

**Affiliations:** ^1^Department of Mental Health and Pathological Addiction, Azienda USL-IRCCS di Reggio Emilia, Reggio Emilia, Italy; ^2^Psychological Emergencies Staff, Azienda USL-IRCCS di Reggio Emilia, Reggio Emilia, Italy

**Keywords:** psychological support, coronavirus pandemic, phone assistance, stress exposure, psychological trauma

## Abstract

We report the field experience of the psychological staff of Azienda USL-IRCCS di Reggio Emilia, a local health system conglomerate serving half a million inhabitants within a catchment area of the Emilia Romagna Region of Italy, during the coronavirus (COVID-19) pandemic. We provided free telephone-based psychological support for the community, with the specific aim of reducing stress caused by the COVID-19 pandemic and its consequences, such as quarantine and lock-down. We describe how the community used this opportunity of psychological support in terms of problems reported and interventions provided. Our field experience suggests that a service of phone psychological support is feasible and quickly implementable in the case of sudden emergencies that affect, to different extents, the whole community.

## Introduction

From the beginning of 2020, the COVID-19 outbreak has become a global pandemic (Zhu et al., 2020) forcing radical measures of public health such as quarantine, physical distancing and lockdowns in several countries, including Italy. Northern Italy in particular was one of the first sub-regions of western Europe hit by the COVID-19 outbreak at the beginning of March, severely stressing the health system (Grasselli et al., 2020).

During this pandemic, healthcare workers, COVID-19 patients and their family members were among those more exposed to stressful events (Duan and Zhu, 2020; Kang et al., 2020) and previous epidemics such as SARS and MERS could be helpful to rapidly identify key issues on immediate and long-term psychological risk for COVID-19 survivors (Mak et al., 2009) and healthcare professionals (Lung et al., 2009; Lee et al., 2018). Moreover, fear of being infected by COVID-19 as well as prolonged quarantine and lock-down could have potential acute and long-lasting psychological *affective* effects also on the community (Brooks et al., 2020; Holmes et al., 2020).

To reduce the potential acute and long-lasting effects of COVID-19 pandemic on mental health, many local health authorities of Northern Italy implemented stepped multilevel and multi-target services of psychological support.

## Context

We report the field experience of the psychological staff of Azienda USL-IRCCS di Reggio Emilia, a local health system conglomerate serving half a million inhabitants in the catchment area of the Emilia Romagna Region of Italy.

A double-level intervention was rapidly implemented in the early weeks of the COVID-19 pandemic emergency. The first level of psychological intervention was provided by hospital-based psychologists usually involved in psycho-oncological support and was directed to healthcare workers, COVID-19 hospitalized patients and family members of patients who died after contracting COVID-19, to alleviate the symptoms and emotional distress induced by disturbing life experiences.

The second level of psychological intervention, that represents the focus of this perspective paper, was provided by psychologists of the Department of Mental Health and Pathological Addictions, that were involved in a service of free phone-based psychological support for the community, with the specific aim of reducing the stress caused by COVID-19 pandemic and its consequences, such as quarantine and lockdown.

Although the use of phone-based methods in psychological counseling and crisis intervention has a long history, especially for some targets as suicidal crisis (Lester, 1977), and its specificity has been widely examined (Lester and Rogers(eds), 2012), its potential in managing psychosocial stress following community trauma exposure has not been consistently reported or investigated (Watson and Hamblen, 2017), especially for global-scale phenomena such as the COVID-19 pandemic. Indeed, the current pandemic scenario invigorated the interest (Zulfic et al., 2020) for the more general issue of telepsychology (American Psychological Association, 2013), in which it is possible to include phone-based psychological support.

All enrolled participants in the project were licensed psychologists/psychotherapists, that underwent a specific online course taught by the head of psychological emergency staff (LT) on the management of psychological reactions induced by emergencies or mass trauma, as post-traumatic symptoms. This course also focused on possible psychological issues raised by the COVID-19 pandemic, as preliminarily reported by the earliest Chinese experiences of COVID-19 (Duan and Zhu, 2020).

Interventions were coded in three steps structuring the psychological support:

– First step: active listening focused on *containment*, i.e., a brief assessment of the stressing situations and related feelings and behaviors.
– Second step: reframing reported symptoms based on strategies of *normalization* (subjective feelings and behaviors as typical reactions to traumatic or severely stressing situations) and/or *psychoeducation* (explanations of typical and physiological reactions to abnormal and acute events, as traumatic events, including intrusive thoughts, hyperarousal, negative mood, avoidance).
– Third step: broad indications on *coping and stress reduction skills*, individualized for children, adults, and elderly people, in case of mild symptoms, or *referring* to other mental health professionals in case of need of more specific interventions. Referring involved hospital psychologists in the case of recently discharged COVID-19 hospitalized patients or family members of dead COVID-19 patients, for rapid psychological support. In other cases, such as acute manifestations of subjective psychological distress, referring involved mental health staff for adequate assessment, support and therapy.

All psychologists were provided with a sheet for each call, that was anonymously filled with basic data (given name, age, living area, stressing situation) and with psychological interventions provided. Phone calls were not recorded and were not used for training scopes or for research (in addition to this report). Supervision was available and provided in case of need by the head of psychological emergency staff (LT), while group sessions of supervisions were not implemented both for lock-down and distancing measures.

The service of psychological phone support was publicized on the local health authority website as well on social media and on local newspapers.

## Results

### General Data

**FIGURE 1:**
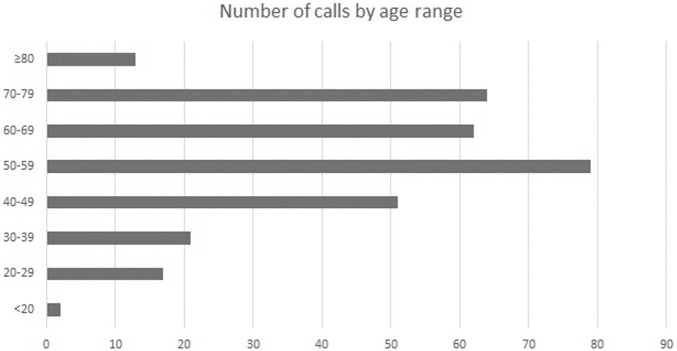
Number of calls by age range.

The service of free phone-based psychological support was active for a total of 11 consecutive weeks since the beginning of the COVID-19 pandemic. During this period, the service received 312 phone calls (227 from females: 72.8%; 85 from males: 27.2%), with a decreasing temporal trend after the initial peak. The mean age of callers was 56.8 (±14.8) years: age distribution of callers is reported in Figure 1. Within callers, 231 (74%) were from the general community, 36 (11.5%) were quarantined patients, 18 (5.8%) were family members of COVID-19 infected or dead patients, and 12 (3.8%) were patients of mental health services.

### In-Depth Analysis of First 3 Weeks

We examined more in depth the first 3 weeks of activity (March 18–April 8), that temporally coincided with the hardest period of COVID-19 management by the health system in Italy. During this period, the psychological staff received 158 phone calls (first week 54, second week 60, third week 44). Phone callers were 41 males (25.9%) and 117 females (74.1%); age ranges of phone callers were <20 years (*n* = 2: 12.7%), 20–29 years (*n* = 6: 3.8%), 30–39 years (*n* = 14: 8.9%), 40–49 years (*n* = 34: 21.5%), 50–59 years (*n* = 34: 21.5%), 60–69 years (*n* = 29: 18.4%), 70–79 years (*n* = 31: 19.6%), ≥80 years (*n* = 8: 5.1%). 149 calls came from the province of Reggio Emilia, while others came from other provinces. The mean duration of calls was 32 min. Most common reasons for calling were the onset or the increase of anxious and psychosomatic symptoms related both to the fear of infection and to the quarantine (95 calls: 60.1%), physical symptoms suggestive of a possible COVID-19 infection (29 calls: 18.4%), preoccupation for other persons such as family members or friends in relation to their COVID-19 infection or isolation due to quarantine (11 calls: 7.0%). Other less prevalent reasons for calling included the possible loss of work, the online schooling of sons, worsening of the affective relationship with spouse, the availability of devices for personal protections (i.e., masks).

As regards psychological interventions, containment was provided in 147 calls (93.0%), normalization in 131 calls (82.9%), psychoeducation in 99 calls (62.7%), coping and stress reduction skills in 129 calls (81.6%) and referring in 59 calls (37.3%). Referral related to COVID-19 infection (discharged hospitalized patients or family members of infected or dead patients) was suggested in 18 calls and 16 subjects contacted hospital psychologists for a specific EMDR-based treatment. Other cases of referral regarded the presentation or the worsening of severe symptoms of psychological distress, such as anxiety, panic attacks or suicidal thoughts.

## Discussion

Help-seeking through phone-based psychological support was more appealing for women, as expected on the basis of empirical evidence (Addis and Mahalik, 2003) and for subjects with middle or advanced age (<40 years 13.9% of callers, ≥40 years 86.1% of callers), with the latter characteristic probably due to the different perceived risk in case of COVID-19 infection on the basis of age.

The majority of themed issues during the first 3 weeks of activity mainly involved current psychological and psychosomatic reactions related to the COVID-19 pandemic, because this period temporally coincided with the hardest moment for health systems in the management of hospitalized infected patients and the highest media coverage. In almost all phone calls, psychological support went through the three steps of intervention, from a preliminary containment based on an active listening of the situation (Lester, 1977; Lester and Rogers(eds), 2012) through a reframing of symptoms with normalization or psychoeducation, to broad indications on strategies to cope with and reduce stress. For example, during the pandemic peak, many isolated or quarantined subjects reported to spend many hours every day listening to news related to the pandemic trend, increasing feelings of anxiety; therefore, in this case it was strongly suggested to reduce exposure to the COVID-19 infodemic (The Lancet Infectious Diseases, 2020). Only in a minority of cases, referring was suggested, due to more severe stress exposure (discharged hospitalized patients or family members of infected or dead patients) or severe symptoms of distress (such as anxiety, panic attacks or suicidal thoughts). During subsequent weeks, in which the number of calls progressively decreased, the main theme of issues shifted from fear of infection and anxiety related to the pandemic to individual psychological difficulties related to quarantine and isolation, with feeling of loneliness especially in oldest subjects living alone. From the perspective of the staff involved in the psychological support, it was a common feeling among psychologists to be more able to respond efficiently to more general themes, as fear of contagion, psychosomatic symptoms or “infodemic-addiction,” while preoccupations related to COVID-19 infection (such as physical symptoms suggestive of infection or delays in specific COVID-19 testing) or feelings related to objective conditions of distress such as prolonged isolation or quarantine were more difficult to manage; for example for some categories of subjects such as elderly patients living alone it was more difficult to suggest coping strategies. Moreover, despite the higher level of anonymity (Lester, 1977; Lester and Rogers(eds), 2012) and the shorter duration of phone sessions in comparison with usual face-to-face sessions (mean duration 32 min), the quality of the relationship established with callers was good in most cases, in line with a recent systematic review on phone psychological therapy (Irvine et al., 2019).

In conclusion, the field experience detailed above suggests that in the case of mass emergencies, health agencies may rapidly activate their own human resources with psychological competencies, to implement a service of phone psychological support to the community. This action is feasible and should be included in specific guidelines to support public health preparedness (Reifels et al., 2013; Watson and Hamblen, 2017).

## Data Availability Statement

All datasets generated for this study are included in the article/supplementary material.

## Ethics Statement

Ethical review and approval was not required for the study on human participants in accordance with the local legislation and institutional requirements. Written informed consent for participation was not required for this study in accordance with the national legislation and the institutional requirements.

## Author Contributions

FG, GG, GMG, LT, and MP conceived the manuscript. MP wrote the first draft. EBe, EBo, FB, SB, FC, KC, FE, VF, RG, JL, RL, AM, GM, SP, AP, CR, MS, VV, AZ, SZ, LP, and MP participated in the project of phone-call psychological support. All authors revised the manuscript and approved the final version.

## Conflict of Interest

The authors declare that the research was conducted in the absence of any commercial or financial relationships that could be construed as a potential conflict of interest.

## References

[B1] Addis M. E. Mahalik J. R. (2003). Men, masculinity, and the contexts of help seeking. *Am. Psychol.* 58 5–14. 10.1037/0003-066x.58.1.5 12674814

[B2] American Psychological Association (2013). *Guidelines for the Practice of Telepsychology.* Avaliable at: https://apa.org/practice/guidelines/telepsychology.aspx (accessed August 09, 2020).

[B3] Brooks S. K. Webster R. K. Smith L. E. Woodland L. Wessely S. Greenberg N. (2020). The psychological impact of quarantine and how to reduce it: rapid review of the evidence. *Lancet* 395 912–920. 10.1016/s0140-6736(20)30460-8 32112714 PMC7158942

[B4] Duan L. Zhu G. (2020). Psychological interventions for people affected by the COVID-19 epidemic. *Lancet Psychiatry* 7 300–302. 10.1016/s2215-0366(20)30073-0 32085840 PMC7128328

[B5] Grasselli G. Pesenti A. Cecconi M. (2020). Critical care utilization for the COVID-19 outbreak in Lombardy, Italy: early experience and forecast during an emergency response. *JAMA* 323 1545–1546. 10.1001/jama.2020.4031 32167538

[B6] Holmes E. A. O’Conner R. C. Perry V. H. Tracey I. Wessely S. Arseneault L. (2020). Multidisciplinary research priorities for the COVID-19 pandemic: a call for action for mental health science. *Lancet Psychiatry* 7 547–560. 32304649 10.1016/S2215-0366(20)30168-1 PMC7159850

[B7] Irvine A. Drew P. Bower P. Brooks H. Gellatly J. Armitage C. J. (2019). Are there interactional differences between telephone and face-to-face psychological therapy? A systematic review of comparative studies. *J. Affect. Disord.* 265 120–131. 10.1016/j.jad.2020.01.057 32090733 PMC7049904

[B8] Kang L. Li Y. Hu S. Chen M. Yang C. Yang B. X. (2020). The mental health of medical workers in Wuhan, China, dealing with the 2019 novel coronavirus. *Lancet Psychiatry* 7:e14 10.1016/s2215-0366(20)30047-x PMC7129673 32035030

[B9] Lee S. M. Kang W. S. Cho A. R. Kim T. Park J. K. (2018). Psychological impact of the 2015 MERS outbreak on hospital workers and quarantined hemodialysis patients. *Compr. Psychiatry* 87 123–127. 10.1016/j.comppsych.2018.10.003 30343247 PMC7094631

[B10] Lester D. (1977). “The use of the telephone in counseling and crisis intervention,” in *The Social Impact of the Telephone*, ed. de Sola Pool I. (Cambridge, MA: MIT Press), 454–472.

[B11] Lester D. Rogers J. R. (eds) (2012). *Crisis Intervention and Counseling by Telephone and Internet*, 3rd Edn Springfield, IL: Charles C. Thomas Publishers.

[B12] Lung F. W. Lu Y. C. Chang Y. Y. Shu B. C. (2009). Mental health symptoms in different health professionals during the SARS attack: a follow-up study. *Psychiatr Q.* 80 107–116. 19247834 10.1007/s11126-009-9095-5

[B13] Mak I. W. Chu C. M. Pan P. C. Yiu M. G. Chan V. L. (2009). Long-term psychiatric morbidities among SARS survivors. *Gen. Hosp. Psychiatry* 31 318–326. 10.1016/j.genhosppsych.2009.03.001 19555791 PMC7112501

[B14] Reifels L. Pietrantoni L. Prati G. Kim Y. Kilpatrick D. G. Dyb G. (2013). Lessons learned about psychosocial responses to disaster and mass trauma: an international perspective. *Eur. J. Psychotraumatol.* 4:10.3402/ejt.v4i0.22897. 10.3402/ejpt.v4i0.22897 PMC3873118 24371515

[B15] The Lancet Infectious Diseases (2020). The COVID-19 infodemic. *Lancet Infect. Dis.* 20 875. 10.1016/S1473-3099(20)30565-X PMC7367666 32687807

[B16] Watson P. Hamblen J. (2017). “Assisting individuals and communities after natural disasters and community traumas,” in *APA Handbooks in Psychology§. APA Handbook of Trauma Psychology: Foundations in Knowledge*, ed. Gold S. N. (Washington, DC: American Psychological Association), 87–97. 10.1037/0000019-005

[B17] Zhu N. Zhang D. Wang W. Li X. Yang B. Song J. (2020). A novel coronavirus from patients with pneumonia in China. *N. Engl. J. Med.* 382 727–733. 31978945 10.1056/NEJMoa2001017 PMC7092803

[B18] Zulfic A. Liu D. Lloyd C. Rowan J. Schubert K. O. (2020). Is telepsychiatry care a realistic option for community mental health services during the COVID-19 pandemic? *Aust. N. Z. J. Psychiatry* 22:0004867420937788. 10.1177/0004867420937788 32571079 PMC7312100

